# Editorial: Characterizing the Multi-Faceted Dynamics of Tumor Cell Plasticity

**DOI:** 10.3389/fmolb.2020.630276

**Published:** 2021-01-20

**Authors:** Satyendra Chandra Tripathi, Mohit Kumar Jolly, Sendurai A. Mani, Herbert Levine

**Affiliations:** ^1^ Department of Biochemistry, All India Institute of Medical Sciences, Nagpur, India; ^2^ Centre for BioSystems Science and Engineering, Indian Institute of Science, Bangalore, India; ^3^ UT MD Anderson Cancer Center, Houston, TX, United States; ^4^ Center for Theoretical Biological Physics, Northeastern University, Boston MA, United States

**Keywords:** cellular plasticity, cancer, epithelial to mesenchymal transition, transcription factor, hybrid states, metastasis, drug resistance, epithelial-mesenchymal plasticity

Cellular plasticity – the ability to dynamically adapt to various changing biochemical and biomechanical, intracellular and extracellular conditions – is a hallmark of cancer aggressiveness. Metastasis, tumor relapse, and resistance against various therapies are manifestations of this multi-faceted phenomenon. Modes of tumor cell plasticity include transitions among phenotypes on the epithelial-mesenchymal spectrum, different subsets of cancer stem cells (CSCs) and non-CSCs, and various metabolic states. These modes depend on a complex interplay between multistable genetic networks, epigenetic regulation and cellular physiology. Furthermore, there is a reciprocal interaction between tumor cell plasticity and plasticity of the microenvironment, as illustrated for example in the alternative polarization states available for macrophages and CD4+ T-helper cells.

Recent in silico, in vitro, and in vivo studies have highlighted that all the processes mentioned above - Epithelial-to-Mesenchymal Transition (EMT), macrophage polarization, and metabolic flexibility related to the Warburg effect - are not binary as originally hypothesized. Instead, cells can acquire a variety of hybrid phenotype(s) with mixed molecular and cellular properties. The hybrid epithelial/mesenchymal (E/M) phenotypes may underpin collective cell migration during metastasis, leading to clusters of circulating tumor cells or emboli, and are typically more ‘stem-like’ and aggressive than cells on either end of the spectrum of this Epithelial-Mesenchymal Plasticity (EMP). Similarly, metabolic phenotypes exhibiting hybrid glycolytic/oxidative phosphorylation processes can drive aggressiveness and therapy resistance. Finally, reversible transitions among non-CSCs and different subsets of CSCs represent a population-level equilibrium maintained among various tumor cell populations.

Progress in charting the underlying regulatory networks mediating these interconnected manifestations of plasticity has facilitated detailed computational studies to identify various nodes in these networks that can potentially serve as biomarkers or therapeutic targets. However, a comprehensive characterization of the dynamics of these transitions and other associated traits such as drug resistance, immune evasion, epigenetic modifications, genetic instability, and cell migration and invasion, and identification of the molecular factors that coordinate these associations, remains incomplete. In this Research Topic, we focus on the molecular and cellular aspects of the multi-dimensional nature of tumor cell plasticity and its implications for cancer progression, metastasis, and tumor relapse. The crucial contributions contained herein cover a broad range of topics related to characterizing the multifaceted dynamics of cellular plasticity.

Sadeghi et al. (2020) performed an integrative analysis to establish the role of EMT within the breast cancer acidic microenvironment. A partial EMT phenotype was observed in the acid-adapted cellular populations, indicating cellular plasticity leading to metastatic competence. The authors also proposed the S100A6 and S100B proteins as key players during the acid-induced EMT phenotypic alteration. Tashireva et al. (2020) categorized molecular subtypes of breast cancer to evaluate stemness and epithelial-mesenchymal transition (EMT) in single tumor cells (STCs). They found that in comparison to mesenchymal-like STCs, cells with epithelial-like morphology more effectively contribute towards breast cancer metastasis. Hellinger et al. (2019) identified an interaction of CYR61 with metastasis-associated protein S100A4 in invasive breast cancer cells. Their findings suggest that inhibiting EMT induced CYR61 reduces ERK1/2 phosphorylation, thereby suppressing S100A4 and hence invasiveness in mesenchymal-ransformed breast cancer cells. In a breast cancer case report, Ruan et al. (2019) focused on a rare pathological phenomenon called cell cannibalism. The authors reported a high frequency of cell-in-cell (CIC) structures with considerable heterogeneity associated with active cell proliferation and poor prognosis. Teo et al. (2020) utilized the 4T1 murine model of TNBC to reveal that Inhibitor of Differentiation Protein (ID1) is expressed in rare neoplastic cells within ER-negative breast cancers. They unveiled a novel mechanism where ID proteins negatively regulate Robo1, activating a Myc transcriptional program. Thong et al. (2020) used single-cell RNA-sequencing to quantify cell state distributions and hybrid stem cell states of the normal mammary (NM) gland throughout the developmental stages of breast and cancer. Their analysis highlighted the phenotypic plasticity of normal mammary stem cells, where E/M hybrids are the most developmentally immature type and play an important role in mammary morphogenesis. Among the breast cancer subtypes, basal tumors expressed a distinct developmentally immature signature.

Cao et al. (2020) identified LOXL2 as a therapeutic target in cervical carcinoma where it is positively correlated with EMT phenotype. They showed that LOXL2 silencing inhibits the proliferation and migration of cancer cells. Panchy et al. (2019) provided insights into the mechanistic basis of cancer cell heterogeneity and plasticity. They combined cancer cell transcriptomics from time course data of EMT in non-tumorigenic epithelial cells, and from epithelial cells with perturbations of key EMT factors in order to perform an integrative analysis. They noticed a wide distribution of cancer cells spread across the EMT spectrum, with ZEB1 playing a key role. Ramirez et al. (2020) used a bioinformatical approach combined with mathematical modelling to analyze a time-series of single-cell RNA-sequencing data of EMT induced cancer cell lines. They constructed common context-specific EMT gene regulatory circuits and identified transcriptional regulators contributing to drive or reverse EMT.

In addition to these original research contributions, the collection of articles also included comprehensive research articles on various axes of plasticity. Kong et al. presented an overall picture of cellular plasticity mediated by the MAPK, PI3K, STAT3, Wnt, Hedgehog, and Notch pathways during breast cancer progression. Drapela et al. discussed the role of ZEB1, a key EMT-inducing transcription factor involved in cell plasticity, response to DNA damage, and in enabling resistance to various therapies. Similarly, Sundararajan et al. (2019) reviewed the roles of GRHL2, an evolutionarily conserved regulator of the epithelial phenotype. Sterneck et al. investigated the respective roles of SLUG, a mediator of partial EMT, and E-cadherin. Zhan et al. (2019) reviewed the role of Asporin in various cancers including breast, colorectal, and pancreatic cancer, where it modulates the EMT transition and hence the migration and invasiveness of tumor cells. After reviewing the increasing amount of evidence for the significance of partial EMT states, Bhatia et al. also discussed clinical developments in targeting epithelial-mesenchymal plasticity (EMP). Clinical and therapeutic implicates of EMP are becoming increasingly crucial, thus indicating that the therapeutic window in the context of EMP needs to be investigated carefully. One proposed idea can be to ‘fix the cells’ on their position of the EMP axis, which may be possible by breaking feedback loops embedded in a cancer cell circuitry (Williams et al., 2019; Hari et al., 2020). Another recent approach can be re-differentiation of cancer cells into normal epithelial cells. Recent approaches include the forced trans-differentiation of EMT-derived breast cancer cells to being adipocytes (Ishay-Ronen et al., 2019).

Beyond EMP, plasticity along the axes of cancer cell stemness was discussed by Thankamony et al. and plasticity along neuroendocrine prostate cancer was highlighted by Tiwari et al. The association of tumor plasticity with senescence-associated pro-inflammatory cytokines was reviewed by Vernot et al. and with mitochondrial involvement was discussed by Denisenko et al. (2019). Finally, non-cell-autonomous mechanisms of cell plasticity i.e., tumor-host interactions, were reflected upon by Mu et al. (2019) focusing on mechanisms related to pancreatic cancer progression. The increasing realization of the plasticity of the TME and especially of its immune components is one of the critical topics ripe for future investigations. We hope you enjoy this series of articles and recognize that the concept of cell plasticity is engendering a revolution in how we think about cancer and how we might be able to create robust interventional strategies.

## Author Contributions

ST and MJ conceived, wrote, and edited the final version of this editorial. SM and HL edited and approved the final version of this editorial.

## Conflict of Interest

The authors declare that the research was conducted in the absence of any commercial or financial relationships that could be construed as a potential conflict of interest.
